# Management and outcome of bleeding pseudoaneurysm associated with chronic pancreatitis

**DOI:** 10.1186/1471-230X-6-3

**Published:** 2006-01-11

**Authors:** Jun-Te Hsu, Chun-Nan Yeh, Chien-Fu Hung, Han-Ming Chen, Tsann-Long Hwang, Yi-Yin Jan, Miin-Fu Chen

**Affiliations:** 1 Department of General Surgery En Chu Kong Hospital 399, Fuhsing Rd, San-shia Town, Taipei Hsien 237, Taiwan; 2 Department of General Surgery, Chang Gung Memorial Hospital, 5, Fushing Street, Kweishan Shiang, Taoyuan, Taiwan; 3 Department of Radiology, Chang Gung Memorial Hospital, 5, Fushing Street, Kweishan Shiang, Taoyuan, Taiwan

## Abstract

**Background:**

A bleeding pseudoaneurysm in patients with chronic pancreatitis is a rare and potentially lethal complication. Optimal treatment of bleeding peripancreatic pseudoaneurysm remains controversial. This study reports on experience at Chang Gung Memorial Hospital (CGMH) in managing of bleeding pseudoaneurysms associated with chronic pancreatitis.

**Methods:**

The medical records of 9 patients (8 males and 1 female; age range, 28 – 71 years; median, 36 years) with bleeding pseudoaneurysms associated with chronic pancreatitis treated at CGMH between Aug. 1992 and Sep. 2004 were retrospectively reviewed. Alcohol abuse (n = 7;78%) was the predominant predisposing factor. Diagnoses of bleeding pseudoaneurysms were based on angiographic (7/7), computed tomographic (4/7), ultrasound (2/5), and surgical (2/2) findings. Whether surgery or angiographic embolization was performed was primarily based on patient clinical condition. Median follow-up was 38 months (range, 4 – 87 months).

**Results:**

Abdominal computed tomography revealed bleeding pseudoaneurysms in 4 of 7 patients (57%). Angiography determined correct diagnosis in 7 patients (7/7, 100%). The splenic artery was involved in 5 cases, the pancreaticoduodenal artery in 2, the gastroduodenal artery in 1, and the middle colic artery in 1. Initial treatment was emergency (n = 4) or elective (n = 3) surgery in 7 patients and arterial embolization in 2. Rebleeding was detected after initial treatment in 3 patients. Overall, 5 arterial embolizations and 9 surgical interventions were performed; the respective rates of success of these treatments were 20% (1/5) and 89% (8/9). Five patients developed pseudocysts before treatment (n = 3) or following intervention (n = 2). Pseudocyst formation was identified in 2 of the 3 rebleeding patients. Five patients underwent surgical treatment for associated pseudocysts and bleeding did not recur. One patient died from angiography-related complications. Overall mortality rate was 11% (1/9). Surgery-related mortality was 0%.

**Conclusion:**

Angiography is valuable in localizing bleeding pseudoaneurysms. In this limited series, patients with bleeding pseudoaneurysms associated with chronic pancreatitis treated surgically seemingly obtained good outcomes.

## Background

A bleeding pseudoaneurysm in patients with chronic pancreatitis is a rare yet lethal complication. Mortality rates can reach as high as 40% depending on patient clinical status, site and characteristics of the bleeding lesion, and the surgical procedure employed [1]. Optimal treatment of bleeding pancreatic pseudoaneurysm associated with chronic pancreatitis remains controversial. Previous studies confirmed the effectiveness of arteriographic embolization for temporary and definite control of bleeding from pseudoaneurysms associated with chronic pancreatitis [2-4]. Some authors have argued that embolization does not cure a diseased pancreas and subsequent surgery is always indicated [5-7]. No evidence-based guidelines exist regarding the optimal treatment modality as limited data is available. This study describes the experience at Chang Gung Memorial Hospital (CGMH) in managing 9 patients diagnosed with bleeding pseudoaneurysms associated with chronic pancreatitis. The medical records of these patients over a period of 12 years were retrospectively reviewed.

## Methods

Between Aug. 1992 and Sep. 2004, 1049 patients with chronic pancreatitis were admitted to the CGMH, Lin-Kou Medical Center, Taoyuan, Taiwan. Nine (8 males and 1 female; age range, 28 – 71 years; median, 36 years) had bleeding pseudoaneurysms. Chronic pancreatitis was defined based on the criteria proposed in Marseille in 1984 [8]. Diagnoses of bleeding pseudoaneurysms was based on angiographic, computed tomographic, ultrasound, and surgical findings. Surgery or angiographic embolization was utilized to treat bleeding pseudoaneurysms depending on the patient's clinical condition. Death during hospitalization or within 30 days was the definition of mortality utilized. Median follow-up was 38 months (range, 4 – 87 months).

### Surgical intervention

Seven of the 9 patients initially underwent surgery. Four of these patients received emergency operations due to a hemoperitoneum (case 1 and 2) with hemodynamic instability, uncontrolled gastrointestinal bleeding (case 7) and acute abdomen (case 9). Three patients underwent elective surgery. Re-operation was required in 2 patients (case 6 and 7) for recurrent pseudocysts. One patient (case 3) underwent subsequent surgery to control bleeding following embolization. Surgical procedures were pseudoaneurysm resection followed by arteriorrhaphy, total gastrectomy, total pancreatectomy, partial pancreatectomy, splenectomy, ligation of bleeder and pseudocyst drainage.

### Angiographic embolization

Transfemoral visceral angiography and embolization were performed by experienced radiological interventionalists. The preferred technique of super selective embolization was employed with stainless steel coils placed distal and proximal to the arterial wall defect or within the aneurysm itself. Coils were placed using a coil pusher or via a flushing method.

In this series, angiographic embolization was performed as the first treatment depending on the patient's general clinical condition, patient's will and site of pseudoaneurysm. In cases with hemodynamic instability, patients underwent emergency surgery. In cases of rebleeding after initial intervention, arteriographic embolization was used as the first-line treatment modality. Angiographic hemostasis was successful when it achieved cessation of bleeding and no rebleeding was observed during follow-up.

## Results

### Patient characteristics

The predisposing factors of chronic pancreatitis were alcohol abuse in 7 patients, pancreas divisum in 1, and idiopathic in 1. Six patients underwent abdominal surgery prior to this study. The interval time from diagnosis of chronic pancreatitis to bleeding pseudoaneurysm onset ranged from 3 months to 4 years (median, 2.4 years). All patients had abdominal pain at admission. Gastrointestinal tract bleeding was detected in 4 patients and hemoperitoneum in 3 (Table 1). Evidence of hemorrhaging with severe or rapidly worsening anemia prompted emergency surgery in 4 patients. Initial treatment was emergent (n = 4) or elective (n = 3) surgery in 7 patients and arterial embolization in 2. Subsequent management entailed embolization in 3 patients, surgery in 3, and radiological intervention in 1 (Table 2).

### Imaging studies

**Figure 1:**
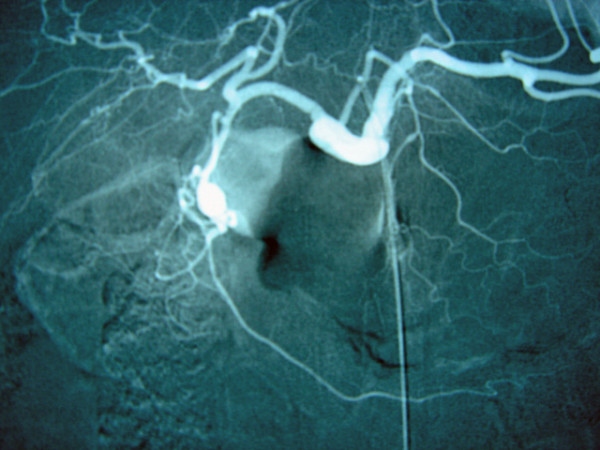
Celiac angiography from case 5 shows a smooth saccular outpouching at the distal gastroduodenal artery, compatible with a pseudoaneurysm.

Abdominal ultrasound showed pseudoaneurysms in 2 of 5 patients (40%). Abdominal computed tomography determined lesions in 4 of 7 patients (57.1%). Angiography obtained correct diagnosis in 7 patients (7/7, 100%). The splenic artery was involved in 5 cases, pancreaticoduodenal artery in 2, gastroduodenal artery in 1 (Figure 1), and middle colic artery in 1. Pseudocysts were detected preoperatively in 3 patients (cases 7, 8 and 9).

### Management and outcome

Table 2 summarizes the evaluation results and outcomes for the 9 patients with bleeding pseudoaneurysms. Seven patients initially underwent surgery for bleeding pseudoaneurysms; 2 of these received operations without a preoperative diagnosis of a bleeding pseudoaneurysm. Embolization was performed as the initial treatment in 2 patients who experienced rebleeding 1 month and 5 months later, respectively. Rebleeding control was successful in 1 patient treated by re-embolization (case 4) and 1 patient treated surgically (case 3).

**Figure 2:**
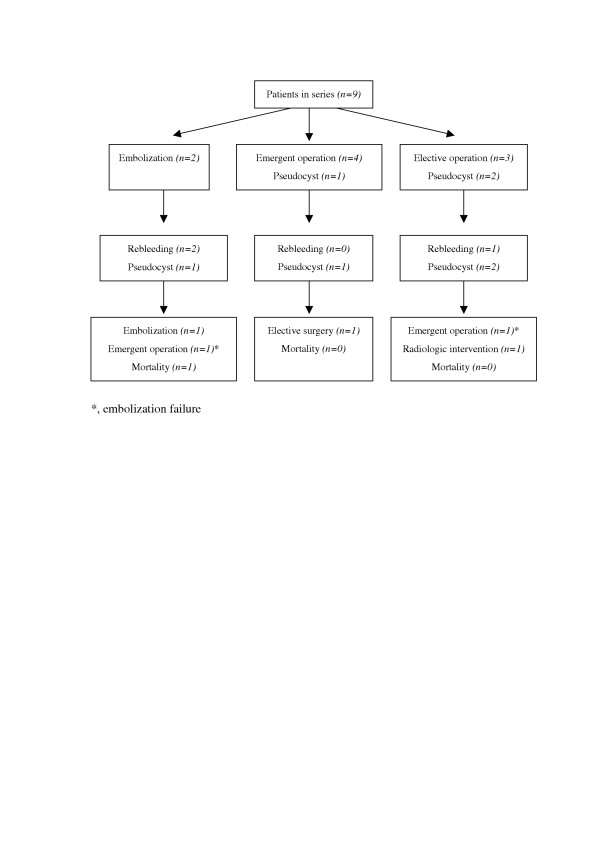
Flow chart showing management and outcome in relation to bleeding pseudoaneurysm treatment.

Two patients developed recurrent pseudocysts after surgery; 1 (case 7) underwent gastrocystostomy for persistent abdominal pain; the other (case 8) received revision for a drainage tube due to occlusion detected by a radiologist. No recurrent bleeding or pseudocysts were observed in these 2 patients. Two patients developed pseudocysts following embolization (case 3) and surgery (case 6) at 1 month and 6 weeks, respectively; both rebled and were treated successfully with surgery after embolization failure. Overall, bleeding pseudoaneurysms were treated with 5 arterial embolizations and 9 operations and success rates were 20% (1/5) and 88.9% (8/9), respectively. One patient (case 3) died from sepsis following treatment for an angiographic complication (bleeding of the right external iliac and femoral arteries). No surgery-related mortality existed. Figure 2 shows a flow chart of management and outcomes in relation to bleeding pseudoaneurysms treatment.

## Discussion

Gastrointestinal bleeding complications in chronic pancreatitis are frequently attributed to peptic ulcer, erosive gastritis, or esophageal and gastric fundus varices. A bleeding pseudoaneurysm is a serious and rare complication of chronic pancreatitis. Consequently, randomized trials for assessing the relative benefits of operative *vs. *radiologic intervention are difficult. Clinicians therefore must rely on data available from observational studies – such that provided in this study.

The following three mechanisms account for pseudoaneurysms related to pancreatitis: 1) severe inflammation and enzymatic autodigestion of a pancreatic or peripancreatic artery producing arterial disruption with pseudoaneurysm formation; 2) an established pseudocyst eroding into a visceral artery, thereby converting the pseudocyst into a large pseudoaneurysm; and 3) a pseudocyst eroding the bowel wall with bleeding from the mucosal surface [5,9]. The splenic artery is most commonly involved, followed by the gastroduodenal, pancreaticoduodenal, and hepatic arteries [1].

Pseudoaneurysms can rupture into the gastrointestinal tract, peritoneal cavity, retroperitoneum, biliopancreatic ducts or pseudocysts. (bleeding into the biliopancreatic ducts is known as hemobilia or hemosuccus pancreaticus.) Clinically, a bleeding pseudoaneurysm typically manifest as silent anemia with melena or as intermittent massive bleeding into the gastrointestinal tract or abdominal cavity, both of which require emergency laparotomy.

Early localization of a pseudoaneurysm via imaging studies is crucial to further treatment. Ultrasound is of little diagnostic value for a bleeding pseudoaneurysm. A dynamic bolus computed tomographic scan is a useful noninvasive approach for detecting pseudoaneurysms [6,10] and associated pseudocysts [11]. However, small pseudoaneurysms can escape detection. In the past 2 decades, angiography has improved its diagnostic accuracy for pseudoaneurysms [1,2,4,12]; in this series angiography reach a sensitivity rate of 100% (7/7). Two patients (22%) in this series underwent laparotomy without pre-operative diagnosis of a bleeding pseudoaneurysm. One patient (case 2) received emergency laparotomy without prior to surgery. The remaining patient (case 8) presented with a huge pseudocyst with abdominal pain; diagnosis of a bleeding pseudoaneurysm was made during surgery. Heightened clinical suspicion in patients with chronic pancreatitis with acute abdominal pain and signs of acute bleeding in combination with appropriate diagnostic modalities is mandatory for early and precise diagnosis.

The reported success of embolization is 79–100% and the reported mortality rate after embolization is 12–33% in patients with acute or chronic pancreatitis [2,3,6,13]. The etiology, pathophysiology, clinical presentation, and treatment and prognosis of hemorrhagic complications for acute necrotizing or chronic pancreatitis differ. Reporting the bleeding complications associated with the 2 categories of pancreatitis together can result in confusion in treating bleeding pseudoaneurysms. In this series, 2 patients initially treated by embolization and 3 patients with rebleeding undergoing subsequent embolization had an overall treatment success rate of 20% (1/5) and a mortality rate of 20% (1/5). The low success rate of embolization in this series may be in part explained due to the definition for successful embolization in this study. Angiographic hemostasis was defined as successful when bleeding stopped with no rebleeding during follow-up; this definition differs from the definition in other series – no rebleeding within 48 hours after embolization [1]. The selection bias and the small number of cases in this study may likely also influence outcomes for embolization.

Some authors consider surgery as the treatment of choice for bleeding pseudoaneurysms in patients with chronic pancreatitis [5-7]. This opinion is supported by this series in that of the 9 surgical interventions to treat bleeding pseudoaneurysms, only 1 patient (1/8, 12.5%) rebled and no patient died because of surgery. By contrast, angiographic embolization as the treatment modality for bleeding pseudoaneurysms resulted in a rebleeding rate of 66.7% (2/3). These observational results can be explained partly by the relatively young age in patients receiving surgical treatment in this series (median age, 35 years). Nevertheless, the small number of patients in this study precludes a comparison of results between surgical intervention and arteriographic embolization.

Debate still exits about the best surgical procedure for treating bleeding pancreatic pseudoaneurysms. Some researchers suggest that proximal and transcystic ligation of a bleeding vessel with internal or external drainage of the cyst is superior to pancreatic resection [14,15]. Conversely, others have suggested that pancreatic resection should be employed as it is the only certain way of preventing the very common problem of rebleeding [2,6,16]. In this study, a pancreatectomy was performed in 4 patients, drainage of pseudocyst in 4, arterial ligation in 2, and resection of pseudoaneurysm followed by arteriorrhaphy in 2 (Table 2). Only 1 patient rebled because of pseudocyst formation after distal pancreatectomy. We suggest that surgical treatment for bleeding pseudoaneurysms should be individualized.

Location of a pseudoaneurysm is a major issue when selecting a treatment course (arterial ligation or resection of the diseased pancreas) and is related to patient outcome. Distal pancreatectomy and splenectomy should be employed to treat bleeding lesions located in the pancreatic tail as these procedures have low morbidity and mortality rates [5,15,16]. Pancreaticoduodenectomy should be limited to select situations and to patients for which less invasive procedures are not technically feasible [5]. Patient outcome is better for patients with lesions in the pancreatic body and tail (mortality, 16%) than for those with lesions in the pancreatic head (mortality, 43%) [17]. In this series, bleeding pseudoaneurysms were located at the pancreatic head in 3 patients. In these patients who were treated by a total of 2 surgical procedures and 4 embolizations, the mortality rate was 33.3% (1/3) and the success rate for controlling bleeding was 33.3% (1/3). In contrast, the remaining 6 patients, whose lesions were located in the pancreatic body and tail and were surgically treated, had an 83.3% (5/6) rate of bleeding control and no mortality.

Several factors can increase risk of acute bleeding in chronic pancreatitis: duration of disease; proximity of a vessel to a pseudocyst; communication with the bile or pancreatic duct; and, splenic vein occlusion from thrombosis [18]. This observation underscores the importance of treating unresolved or progressively enlarged pseudocysts associated with chronic pancreatitis prophylactically to prevent life-threatening bleeding [19]. Because chronic pancreatitis is an ongoing inflammatory process, operative intervention should be performed as soon as possible [6,13]. In this study, pseudocysts occurred either prior to treatment (n = 3) or following intervention (n = 2) in 5 patients; rebleeding pseudoaneurysms developed in 2 of these cases. Drainage procedures were employed in 4 patients, none of whom rebled during the follow-up period (range, 4 – 87 months).

## Conclusion

Management of bleeding pseudoaneurysms remains challenging for clinicians. Angiography is valuable in localizing bleeding pseudoaneurysms. In this limited series, patients with bleeding pseudoaneurysms associated with chronic pancreatitis treated by surgery appeared to achieve good results.

## Competing interests

The author(s) declare that they have no competing interests.

## Authors' contributions

Hsu JT: planning, data collection, study design and analysis, surgical management of patients, drafting and revising the manuscript

Yeh CN: study design and analysis, surgical management of patients, revising the manuscript

Hung CF: radiological management of patients, revising the manuscript

Chen HM: planning, study design and analysis, surgical management of patients, revising the manuscript

Hwang TL: study design and analysis, surgical management of patients

Jan YY: study design and analysis, surgical management of patients

Chen MF: study design and analysis, surgical management of patients

## Pre-publication history

The pre-publication history for this paper can be accessed here:



## Figures and Tables

**Table 1:** Data for the 9 patients with bleeding pseudoaneurysms due to chronic pancreatitis

Case/sex/age (years)	Predisposing factors	History of abdominal operation	Clinical presentation	Site of bleeding
1/M/59	Alcohol	Cholecystectomy	Pain, hemoperitoneum	MCA
2/M/33	Alcohol	Splenectomy	Pain, hemoperitoneum	SA
3/M/46	Alcohol	No	Pain, hemoperitoneum	PDA
4/F/71	Pancreas divisum	Puestow's procedure	Pain, hematemesis	PDA
5/M/36	Idiopathic	No	Pain, melena, hematemesis	GDA
6/M/33	Alcohol	Splenectomy	Pain, melena, hematemesis	SA
7/M/28	Alcohol	No	Pain, melena, hematemesis	SA
8/M/38	Alcohol	Subtotal gastrectomy	Pain*	SA
9/M/35	Alcohol	Subtotal gastrectomy	Pain*	SA

MCA, middle colic artery; SA, splenic artery; PDA, pancreaticoduodenal artery; GDA, gastroduodenal artery

* The pseudoaneurysm ruptured into the pseudocyst

**Table 2:** Management of, and results for, bleeding pseudoaneurysms in the 9 patients with chronic pancreatitis

Case/sex/age (years)	Initial treatment	Associated pseudocyst	Rebleeding	Secondary treatment	Length of follow-up (months)	Outcome
1/M/59	A	No	No	No	57	S
2/M/33	TG, L	No	No	No	38	S
3/M/46	E	Yes*	Yes	E^‡^, L, ED	4	D
4/F/71	E	No	Yes	E	18	S
5/M/36	Nearly TP, Sp	No	No	No	50	S
6/M/33	DP^+^	Yes*	Yes	E^‡^, L, ED	87	S
7/M/28	DP, Sp	Yes^+^	No	GC^+^	15	S
8/M/38	A, ED	Yes^+^	No	RD	51	S
9/M/35	DP, Sp	Yes	No	No	10	S

A, resection of pseudoaneurysm and arteriorrhaphy; TG, total gastrectomy; TP, total pancreatectomy; Sp, splenectomy; DP, distal pancreatectomy; E, embolization; L, arterial ligation; ED, external drainage; GC, gastrocystostomy; RD, revision of drained tube; S, survived; D, died

*, after intervention; +, recurrent, ‡, embolization failure
